# Lipid Droplet, a Key Player in Host-Parasite Interactions

**DOI:** 10.3389/fimmu.2018.01022

**Published:** 2018-05-23

**Authors:** Adriana Lima Vallochi, Livia Teixeira, Karina da Silva Oliveira, Clarissa Menezes Maya-Monteiro, Patricia T. Bozza

**Affiliations:** Laboratório de Imunofarmacologia, Instituto Oswaldo Cruz, Fundação Oswaldo Cruz (FIOCRUZ), Rio de Janeiro, Brazil

**Keywords:** lipid droplets, lipid bodies, parasite, intracellular pathogen, eicosanoids, inflammation, immune response

## Abstract

Lipid droplets (lipid bodies, LDs) are dynamic organelles that have important roles in regulating lipid metabolism, energy homeostasis, cell signaling, membrane trafficking, and inflammation. LD biogenesis, composition, and functions are highly regulated and may vary according to the stimuli, cell type, activation state, and inflammatory environment. Increased cytoplasmic LDs are frequently observed in leukocytes and other cells in a number of infectious diseases. Accumulating evidence reveals LDs participation in fundamental mechanisms of host-pathogen interactions, including cell signaling and immunity. LDs are sources of eicosanoid production, and may participate in different aspects of innate signaling and antigen presentation. In addition, intracellular pathogens evolved mechanisms to subvert host metabolism and may use host LDs, as ways of immune evasion and nutrients source. Here, we review mechanisms of LDs biogenesis and their contributions to the infection progress, and discuss the latest discoveries on mechanisms and pathways involving LDs roles as regulators of the immune response to protozoan infection.

## Introduction

Lipid droplets (LDs) are dynamic and complex organelles, composed of a hydrophobic core enriched in neutral lipids such as triglyceride, cholesteryl ester, and retinyl ester, a surrounding monolayer of phospholipid, cholesterol, and a varied array of associated proteins with diverse functions in cell homeostasis, metabolism, and signaling (1, 2). The accumulation of lipids within the cytoplasmic LDs is a highly conserved feature, due to its role in energy homeostasis, membrane synthesis and cell signaling, conferring an evolutionary advantage to the organism. Indeed, LDs are present in virtually all cell types and organisms, including in unicellular protozoan parasites (3, 4), and in leukocytes and other cells involved in immune response in mammalians (5, 6).

Interest in LDs biogenesis and functions has dramatically increased in recent years due to their association with metabolic and inflammatory diseases, including diabetes (7), atherosclerosis (8, 9), obesity, cancer (10, 11), allergy (12), neurodegenerative diseases (13, 14), and numerous infectious diseases [reviewed in Bozza et al. (15), van der Meer-Janssen et al. (16), and Saka and Valdivia (17)]. Increased numbers of LDs in leukocytes and other cells are observed in infectious diseases caused by bacteria, virus, fungus, and protozoan parasites (17–26). LD accumulation in the cytoplasm of distinct host cell types has been reported after infection with *Plasmodium berguei* (27, 28), *Trypanosoma cruzi* (20, 29, 30), *Toxoplasma gondii* (31–34), and *Leishmania* sp. (35–37) and their contributions in disease pathogenesis are starting to be unveilled.

Different signaling pathways have been implicated in LD biogenesis in leukocytes and other cells involved in inflammatory and/or infectious reactions. Although molecular mechanisms that govern LD biogenesis are not fully characterized, the observations gathered so far indicate the involvement of both pathogen and host factors. Moreover, acumulating evidence indicates that infection-driven LD formation involves specific and well-regulated mechanisms that involve innate immune receptors, scavenger receptors, and nuclear receptors dependent pathways (18, 21, 26, 30, 38–42). Newly formed host LDs play key roles in host-pathogen interactions. Of note host LDs triggered during infection are major intracellular domains involved in eicosanoid synthesis with multiple functions in inflammation and immune signaling (18, 20, 21, 26, 30, 43, 44). More recently, parasite LDs were also demonstrated to compartmentalize eicosanoid synthesis with roles in parasite physiology and escape mechanisms (45, 46). In addition to functioning as signaling platforms, host LDs properties in energy storage and metabolic homeostasia may be explored by intracellular pathogens for their survival and growth. Accordingly, different authors showed a close association of LDs with parasite vacuoles (PVs) (20, 21, 33, 34, 47–49). This association may enable the nutrients’ flow between the host and the parasites, which is of crucial importance for parasite metabolism and may contribute to avoid the host response. Besides the acquisition of nutrients and imbalance of host metabolic pathways, the parasites explore many additional biological host responses with impacts to disease pathogenesis.

Here, we will address the mechanisms of formation and functions of LDs in the interactions between protozoan parasites and mammalian host. We will highlight the LDs role in host-pathogen interactions, discussing the crosstalk between pathogen and host cell on the immune response and survival mechanisms during infection.

## LD: A Dynamic Cell Organelle

### The Organelle Complexity

Lipid droplets are roughly spherical structures consisting mostly of triacylglycerols and sterol esters, lacking a delimiting classical bilayer membrane but surrounding by a monolayer of phospholipids and cholesterol and a variety of associated proteins (50, 51). This unique organization defies understanding how occur transport pathways to receive or deliver protein and lipid, once that arrangement does not fit in classical traffic vesicular mechanisms. On the other hand, favors its distinction from other organelles under electron microscope. More than 160 lipid species have been identified in LDs, with important variations in contents according to the cell type. For instance, triglycerides are largely dominant in white adipocytes, whereas sterol esters are more abundant in macrophages and retinyl esters in stellate cells (52–56). LDs associated proteins vary according to the cell and stimulatory condition and contains perilipin family proteins, lipid biosynthetic enzymes, lipolytic enzymes; enzymes involved in signaling and inflammatory mediator synthesis, and membrane-trafficking proteins (55, 57–65). Proteins associate with LDs surface through amphipathic and/or hydrophobic molecular moieties (66–69). Also, some proteins were detected within the hydrophobic core of the LD (59, 60, 70, 71). However, the most intriguing fact about protein content of LDs certainly is the presence of proteins with predicted membrane insertion domains within these organelles (59, 60, 65, 72). Among the proteins associated with LDs are the well-characterized perilipin familys’ proteins that share sequence similarities, previously termed as the PAT family, often used as molecular markers of LDs. It includes perilipin 1, perilipin 2 [PLIN2/adipose differentiation-related protein (ADRP)], perilipin 3 [tail-interacting protein of 47 kDa (TIP-47)], perilipin 4 (S3-12), and perilipin 5 (LSDA5, LSDP5, MLDP, and OXPAT) (73). Perilipins are major structural proteins present at the surface of LDs involved in regulating LD formation, incorporation, and accumulation of lipid in different cells types (74–79). The enzymes Acyl-CoA: diacylglycerol acyltransferase 2 (DGAT2) and acyl-CoA: cholesterol acyltransferase (ACAT) are responsible for the synthesis of the main LD core lipids (TAGs and CE). DGAT2 is inserted exclusively into the external leaflet of the endoplasmic reticulum (ER) membrane and, therefore, can diffuse onto the LD surface during LD formation, promoting TAGs synthesis and the continuation of LD growth (80). LDs also compartmentalize the lipolysis enzyme, adipose tissue triacylglycerol lipase (ATGL), as well as perilipins, that modulate both hormone-sensitive lipase and ATGL activities (80–82).

Lipid droplets are organelles that are intimately related to the ER (1, 83). The first and still accepted model of LDs formation suggests that these organelles are derived from the accumulation of newly synthesized lipids within the double layer of the ER membrane, with subsequent budding off from the ER to the cytoplasm after reaching a critical size (50, 83). According to this model, newly formed LDs detaches are surrounded by a phospholipid monolayer derived from the cytoplasmic leaflet of the ER coated with proteins that lack transmembrane spawning domains (3, 70, 83–85). However, accumulating evidence, based on imaging and composition of LDs, reported the presence of membrane-associated and transmembrane spanning proteins, as well as ribosomal structures and ribosomal associated proteins, giving rise to new hypothetical models for LD biogenesis (2, 65, 70, 86). These different groups suggested that invaginations of ER inside the LDs could be the membranous structure and hydrophilic ambient that allow proteins in the core of LDs (65, 72, 86), and/or that LDs may remain associated to ER through physical bridges (2, 87). Together, these biogenesis models contemplate the existence of subcompartments inside these organelles made possible through the incorporation of ER-derived membranes enabling sequential enzymatic reactions to occur within LDs as in the case of eicosanoid synthesis (5, 86, 88, 89).

Lipid droplets have gained importance as specialized and inducible cytoplasmic organelle due to their various proteic and lipidic components, and their participation in fundamental cellular processes. LD functions extend beyond the regulation of lipid metabolism in cell signaling, immunological activation, membrane trafficking participation, and control of the synthesis and secretion of inflammatory mediators. The LD in infectious diseases also suggests functions beyond the mere interaction with different viral proteins or bacteria; they can participate in important celular pathways of immune system.

One of the best characterized functions of LDs in leukocytes and other cells of the immune response are their capacity to act as platforms for heithened eicosanoid synthesis (90, 91). Eicosanoids—including prostaglandins, leukotrienes, and lipoxins—are non-storable but promptly newly synthesized signaling molecules, derived from the enzymatic oxygenation of arachidonic acid (AA), that control key cellular processes, including cell activation, metabolism, migration, proliferation, and death (92). Intracellular compartmentalization of eicosanoid synthesis within leukocytes and other cells has emerged as a key feature that regulates the amount and may also regulate the eicosanoid produced, and LDs has been established as main locales involved in the increased eicosanoid synthesis in inflammatory conditions (90). The substrate for eicosanoid synthesis— AA—is present in LDs, esterified in phospholipids and neutral lipids, where it can be converted into eicosanoids by specialized enzymes (6, 55, 93–95). cPLA_2_ and ATGL were shown to co-localize with LDs in different cells where it participates in the release of AA-associated LD from phospholipids and triglyceride pool, respectively (55, 96–98). Notably, *P. aeruginosa* toxin ExoU also mobilizes AA from host LDs due to their phospholipase activity as part of their pathogenic activity (94). The major eicosanoid-forming enzymes—cyclooxygenase, 5-lipoxygenase (5-LO), 15-LO, 5-LO-activating protein, and the terminal enzymes including PGE_2_ synthase and LTC_4_ synthase—were described within LDs of stimulated cells obtained under inflammatory and infectious conditions (18, 21, 26, 30, 43, 44, 59, 60, 99). Increased LD biogenesis correlates with an enhanced capacity of the cells to produce eicosanoids and suggests that the compartmentalization of the eicosanoid-synthetic machinery within LD have roles in the cellular ability to generate eicosanoids (18, 21, 59, 100). Direct proof of eicosanoid synthesis at LDs came only after the development of a lipid immunolabeling technique termed EicosaCell (88, 101). Since eicosanoids are not stored but are synthesized and released immediately, the EicosaCell method enables to immobilize and label eicosanoids at the exact locale of their synthesis, and by the use of this method the synthesis of leukotrienes and prostaglandins occuring at LDs have been confirmed (21, 30, 43, 101). Accordingly, it has been established that LDs are specialized, inducible cytoplasmic domains that have central roles in the control of synthesis and secretion of inflammatory mediators under different pathophysiological conditions, including in infections [reviewed in Bozza et al. (90)].

### Mechanisms of LD Formation in Infectious Diseases Involves Specific and Regulated Mechanisms

Lipid droplet biogenesis in host cells during infections is a highly regulated cellular event. Although still incomplete, great advances on the understanding of the molecular events that govern LD formation in cells during host-pathogen interactions have been achieved in recent years. As detailed below, accumulation of LDs can be observed rapidly after infection with a variety of pathogens, and results from a balance between new lipid synthesis, increased lipid uptake, and regulated lypolisis involving both transcriptional dependent and independent mechanisms. Of note, during infection, not only infected cells and/or cells in direct contact with pathogens exhibit increased accumulation of LDs in their cytoplasm, but also non-infected neighboring cells at the inflammatory microenvironment may also present increased numbers of LDs indicating that paracrine mechanisms of bystander amplification through host and pathogen-derived signals also take place.

Pattern recognition receptors have been found to be involved in the generation of signals that induce LD formation. The main family of pattern recognition receptors with large range capacity of sensing diverse pathogens is the toll-like receptors (TLR). Cells from TLR4-null mutant mice and cells from signaling inactive mice fail to form LD after stimulation by LPS or during experimental sepsis, establishing the dependency of TLR4 for macrophages LD formation (18, 43). TLR4 signaling triggers the production of inflammatory mediators like platelet activator factor (PAF) and MCP-1/CCL2 that amplify the LD-induced response (18, 21, 43). Other proteins found to be required for productive signaling in response to LPS, include LPS-binding protein, CD14, CD11b/CD18, and myeloid differentiation factor-2 because their neutralization inhibits LPS-induced LD formation (18, 43). Indeed, different pathogens may be specifically recognized through innate receptors to trigger LDs in host cells. TLR2-dependent mechanisms have been implicated in LD formation triggered by *Mycobacterium bovis* (BCG), *Mycobacterium leprae, T. cruzi, Chlamydia pneumonia, Histoplasma capsulatum*, and schistosomal-lipids (21, 26, 38, 44, 102, 103). The studies of *M. bovis* (BCG) infection and stimulation by the purified cell wall component lipoarabinomannan highlighted the importance of TLR2 in LD formation, but not essential roles of TLR4 or TLR6 (21, 38, 104). However, classic TLR2 ligands as Pam3Cys and Zymozan and non-pathogenic mycobacteria *Mycobacterium smegmatis* failed to induce LDs formation or PGE_2_ production, while still inducing TLR2-dependent TNF-α production (38). This finding suggests that TLR2 activation, although essential is not sufficient to trigger pathways of LDs formation and other cofactors may be involved. Indeed, CD36, a multi-ligand scavenger receptor, has been demonstrated to cooperate with TLR2 in BCG signaling and LDs biogenesis in a mechanism dependent of compartmentalization within lipid rafts (39). Similarly, in *M. leprae* infection, TLR2 and TLR6 were implicated in LD formation and bystander amplification in macrophages with no internalized bacteria. This suggests an important role for these receptors in LD biogenesis, not only by direct recognition of microbial components, but also by leading to secondary autocrine/paracrine signaling (21, 44). Accordingly, conditioned medium from *M. leprae*-infected wild-type cells induced LD in wild-type and TLR2-deficient cells (40). Notably, many cytokines and chemokines have been described as being able to lead LDs formation (12, 18, 43, 99, 105–107). Indeed, MCP-1/CCL2 has been implicated in sepsis triggered LD formation, whereas IL-10 and CCL3 were shown to participate in mechanisms of mycobacterial LD formation (41, 43). Lipid signaling is also involved in LD formation and may participate in mechanisms of bystandar LD formation. PAF and PAF-like lipids are involved in LPS and oxidized low-density lipoprotein (LDL)-induced LDs formation, since pretreatment with PAF receptor antagonist significantly inhibits the number of these organelles during experimental sepsis (18, 43, 107). In addition, cis-unsaturated fatty acids including AA and oleic acid are potent inducers of LD formation in leukocytes and other cells (106, 108). TLR2 also mediates LD formation during protozoan parasite infection. *T. cruzi* infections induced LD formation in macrophages in a TLR2-dependent manner. Apoptotic cell uptake by these macrophages potentiated the LD formation and PGE_2_ production *via* TGF-β signaling. The inhibition of LD formation by the fatty acid synthase inhibitor C75 reverted the potentiating effects of the apoptotic cells on LD biogenesis, eicosanoid synthesis, and parasite replication, suggesting a role for LD in lipid mediator production and parasite escape mechanisms. Curiously, the effect was not restricted to parasitized cells, suggesting the involvement of paracrine mediators in LD biogenesis (30). Other classes of pattern recognition receptors have also been implicated in LD biogenesis, including dectin-1 (26).

Downstream mechanisms involved in infectious triggered LD formation are starting to be described. In this sense, signaling pathways involved in expression of genes related to influx/efflux of lipids and *de novo* synthesis of lipids during infectious process have been evaluated. Recently, it was reported that bacterial components may modulate expression and function of PPARγ. PPARγ is a member of lipid-activated nuclear receptor family that directly regulates several genes participating in fatty acids uptake, lipids storage, and inflammatory response. PPARγ heterodimerize with retinoid X receptor that binds directly to DNA response elements present in target gene. In macrophages and dendritic cells, PPARγ have proven to be a key regulator of lipid metabolism and inflammatory genes (109). In addition, PPARγ signaling pathway has been identified as central to foam cells formation in atherosclerotic lesions (110–113). During *M. bovis* BCG infection, TLR2 regulates the expression and activation of PPARγ activation as well as LDs formation. PPARγ inhibition by its antagonist significantly reduces number of LDs in BCG-infected macrophages, indicating its involvement in LDs biogenesis during mycobacterial infection (38). PPARγ involvement in LDs biogenesis may occur at different levels. PPARγ regulates expression of genes related to *de novo* lipid synthesis such as fatty acid synthase enzyme and PLIN2/ADRP, increasing TAG and cholesterol storage and reducing cholesterol efflux (114–116). PPARγ can also increase the influx of exogenous lipids due to increased expression of scavenger receptor CD36 (39). By contrast, liver X receptor, whose activation increases cholesterol efflux, has its transcriptional activity inhibited when viral and bacterial antigens are recognize *via* TLR3 and TLR4 receptors (117). Together these data show that LDs formation depends on specific signaling pathways activation, which regulate influx and efflux balance of lipids in cells. *C. pneumoniae* express a variety of potential TLR ligands and induces foam cell formation, facilitating atherogenesis. *C. pneumoniae*-induced LD formation also involves MAPK phosphorylation, including JNK and ERK, participates in macrophage LD formation. *C. pneumoniae* infection induced the down-regulation of PPARα and PPARγ expression (118), in contrary to the upregulation observed in BCG-infected macrophages where upregulation of PPARγ and PPARγ-dependent LD formation was described (39). Surprisingly, JNK and ERK inhibitors blocked LD formation and suppressed the down-regulation of PPARα and PPARγ. PPARα or PPARγ agonists reverted the infection-induced JNK and ERK phosphorylation while PPARα and PPARγ antagonist enhanced these phosphorylations. The increased *C. pneumoniae*-induced LD formation in LDL-treated THP-1 macrophages was interpreted to be the result of crosstalk between MAPK and PPARα/γ signaling pathways (118). Changes in the intracellular concentration of PPAR ligands can influence PPAR-dependent gene regulation (119).

Increasing amounts of data regarding the molecular mechanisms that govern lipogenesis, as well as regulation of the influx and efflux of lipids, suggest that these mechanisms operate synergistically for LD formation during infection (34, 39, 40).

## LDs in Parasite Infections

### Parasite Lipid Homeostasis and Their Own LDs

Throughout the entire infection process beginning with the host cell invasion, there is a huge involvement of lipids from both members’ interactions (120–122). Some parasites build and survive inside their PV; others leave them to live in host cytoplasm. The vacuole protects parasites but complicates access to some essential factors for parasite proliferation as, e.g., lipids. Intracellular parasites scavenge cholesterol from LDL or host LDs. LDs are a vital source of energy, and an essential element for the homeostasis (detoxification) of blood-stage parasites, as *Plasmodium* spp. and *Schistosoma* spp. Intracellular parasites have their own LDs—as an energy storage source, for metabolic homeostasis, and to modulate and escape the host response. The roles of parasites’ LDs and their interplay with host cells will be discussed in the following section.

#### Protozoa Are Unable to Synthesize Cholesterol *De Novo* and May Retrieve It From Host LDs

Despite possessing complex molecular machinery to interact in a variety of ways with their host cell, protozoa are unable to synthesize cholesterol *de novo. T. cruzi* recruits host lysosomes for fusion with the PV to initiate its egress from the PV and enter into the host cell cytoplasm, where replication begins (123). Parasites of the genus *Leishmania* evolved mechanisms to survive and multiply within acidified vesicles enriched in lysosomal enzymes (124). Otherwise, *T. gondii* and *Plasmodium* spp. have a so-called “active invasion,” and both built a nonfusogenic PV. The *T. gondii* PV interacts with many host structures such as LDs, microtubules, endosomes, the ER, mitochondria, and Golgi vesicles, which closely associated with the vacuole for nutrient acquisition (34, 125). *T. gondii* produces a network of tubule-like structures called the intravacuolar tubulovesicular network, a membranous interface derived from multilamellar vesicles secreted by the parasite (126, 127). However, protozoan parasites rely on their ability to steal cholesterol from different sources of the host (128–130).

As in mammals, *T. cruzi* captures cholesterol from LDLs *via* LDL receptor (LDLr). Experiments using LDLr knockout cells showed a 62% decrease in the total parasite burden compared with wild-type *T. cruzi*-infected mice. LDLr signaling is also essential in the invasion and fusion of PV with host lysosomes (129). *T. brucei* also needs LDL or high-density lipoproteins (HDL) for its multiplication (131).

*Leishmania amazonensis* has specific binding sites to HDL and LDL in its membranes. The parasite can capture cholesterol from LDL, and this process involves detergent-resistant membrane lipid microdomains. In all *L. amazonensis* extensions, host cholesterol is found; the intracellular compartments engaged in parasite lipid traffic and the presence of the LDLr remain to be discovered (130). Recently, Carvalho and colleagues found that some components of lipid metabolism had an association with lipoprotein lipase (LPL), apolipoprotein E (ApoE), and PPARα gene polymorphisms and presented the risk markers of visceral leishmaniasis (VL). Individuals with clinical manifestation of VL filed high TAG and very-low-density lipoprotein cholesterol (VLDL-C) levels, and low high-density lipoprotein cholesterol (HDL-C) levels. Furthermore, the LPL mutation (H+/H+ genotype and the H+ allele) was associated with elevated VLDL-C and TAG levels, reduced HDL-C levels and revealed an odds ratio of 21.3. The L162V polymorphism in the PPARα gene showed an odds ratio of 8.77 for Leu/Val when compared with Leu/Leu genotype. Thus, high TAG and VLDL-C levels were associated with susceptibility to VL, whereas low HDL levels with resistance to *L. infantum* infection. The mutated LPL and the PPARα Leu/Val genotypes may be considered risk markers for the development of VL (132).

*Trypanosoma cruzi* infection is capable of modulating cholesterol metabolism in the host tissue in both acute and chronic patients (133) as well as *L. major* in mice macrophages (48). Quantitative PCR analysis shows an 8,000-fold increase of LDLr levels in heart tissue of *T. cruzi*-infected mice compared with the control. Both, the accumulation of LDL and its receptor were observed around amastigote nests within infected cardiomyocytes (129). Like *T. cruzi, T. brucei*, and *L. amazonensis, T. gondii* also scavenges cholesterol from plasma (128). Experiments using LDLr knockout cells showed a 45% reduction in parasite load in *T. gondii*-infected LDLr knockout mice compared with control mice (134). However, Milovanović and colleagues showed a decrease in HDL and cholesterol in *T. gondii*-infected outbred mice, but LDL was unaltered until day 42 of infection. Moreover, they only observed an increase in LDL and consistent triglyceride levels at this end-point. The parasitemia only correlated with HDL levels and with LDL when the LDL levels were above 300 (135).

Host LDs are sources of lipids for many intracellular parasites. Intracellular parasites have evolved ways to attract host LDs to their phagosomal compartment and engulf them intact by unknown mechanisms (20, 33, 34, 48, 49). Although the *Toxoplasma* PV shares no characteristics with a phagosome, *Toxoplasma* also internalizes host LDs into the PV lumen. In *Toxoplasma*-infected cells, this process is independent of host Golgi or endoplasmatic reticulum, and the parasite has cholesterol equally distributed, but the PV membrane is low in cholesterol. When in excess, parasites release cholesterol and phosphatidylcholine using membrane-associated ABC-transport proteins (136). Overexpressing TgRab51, a Rab5 homolog, parasite mutants have increased numbers of LD by enhanced uptake of cholesterol and had their growth accelerated (137).

**Figure 1:**
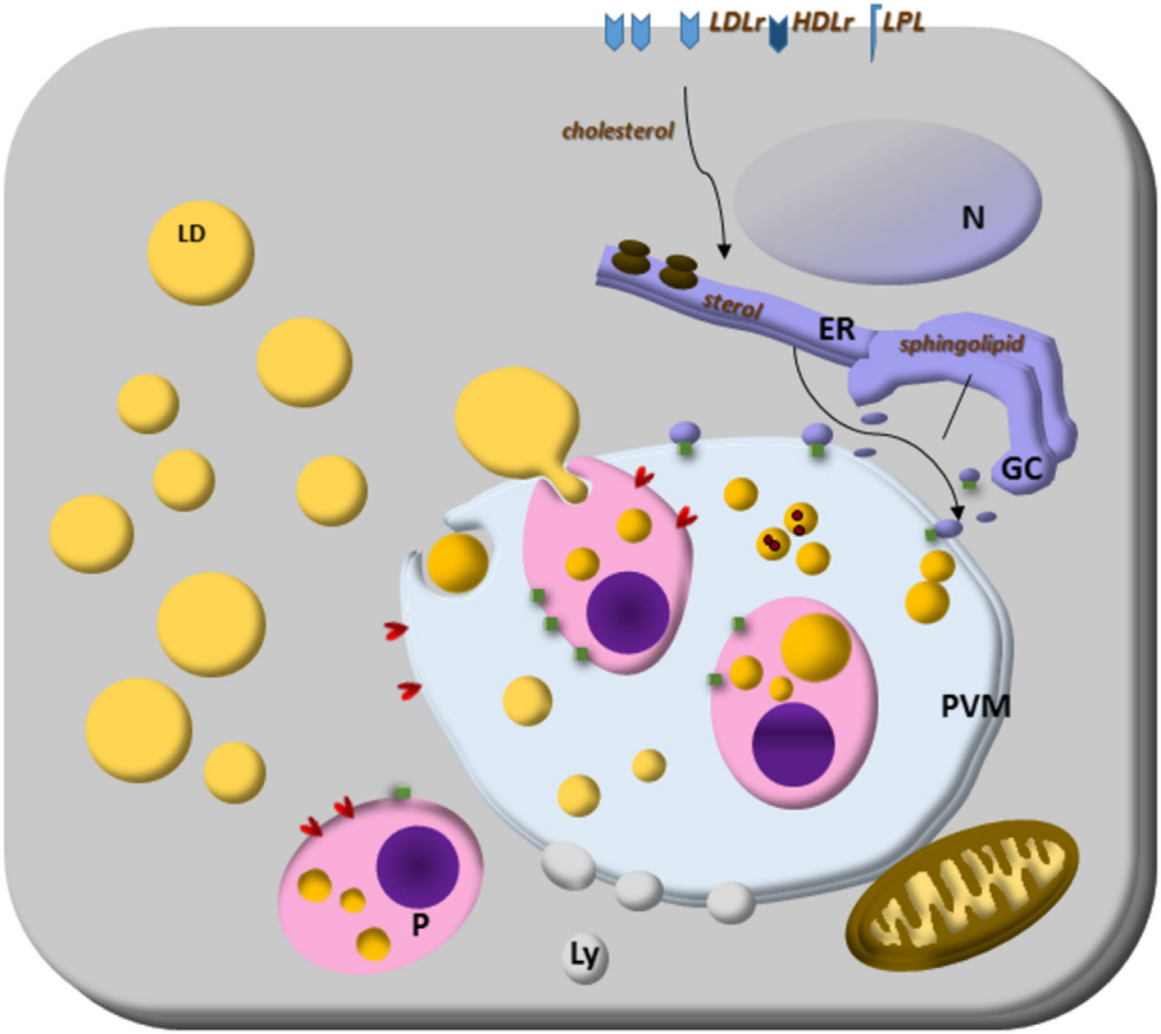
Lipid droplets (LDs) on parasites lipid homeostasis. *Trypanosoma* spp., *Leishmania* spp., *Plasmodium* spp., and *Toxoplasma gondii* cannot synthesize cholesterol, and the energy and lipid source for them is the host lipid synthesis and host LDs, as well as low-density lipoproteins, eventually high-density lipoproteins particles and lipoprotein lipase. The close relationship with other organelles through Rab proteins can be lipid sources, such as endoplasmic reticulum (ER) for sterol and Golgi complex (GC) for sphingolipid sources. Although the mechanisms are still unknown, some parasites can store lipids on their LD. Lipoprotein- and ABC-proteins are involved in the mammalian lipid transport, as well as important enzymes to lipid synthesis like ACAT and DGAT proteins. These proteins are also present in *T. gondii* and ACAT-like proteins are present in *Trypanosoma cruzi*. The parasite transfer lipids to their own LD to be stored, but in *Plasmodium* spp., or they metabolize them as in *Plasmodium* and *T. gondii*. The parasite and host LDs are involved in essential cell functions that can favor the survival of the parasite, such as energy and lipid source for intracellular parasites proliferation. They also can isolate lipids or toxic metabolites from the cytoplasm for detoxification, such as hemozoin from heme on *Plasmodium*-infected cell LD.

*Toxoplasma gondii* scavenges host LDs’ cholesterol and their lipolytic enzymatic activities to grow for its infectibility. It intercepts and engulfs host Rab7-associated LDs to the PV lumen, and the parasite lipase releases lipids from host LD making them accessible to the parasite. These LDs can be captured by endocytosis (34) and the parasite can modify the host-derived lipids (138), largely exploring the host lipidome (34). Conversely, *Plasmodium* cannot store cholesterol, because they do not express an ACAT enzyme, indicating that the parasite needs to scavenge lipids from the host constantly (139). The blood-stage form of *Plasmodium* scavenges cholesterol from HDL particles or directly from the erythrocyte membrane; how cholesterol is then transported to the parasite is still unknown. It is important to note that *Plasmodium* expresses sterol translocators and transporter genes and they can collect membrane components in their plasma membrane (140, 141). Furthermore, the *Plasmodium* PV has physical contact with the host plasma membrane, and the intracellular parasites can access cholesterol (142). *Plasmodium* scavenges cholesterol from LDL and HDL particles and LDs from hepatocytes. *Plasmodium*-infected hepatocytes have genes encoding sterol biosynthesis and metabolism components upregulated (143). Both LDL-derived and host synthesized cholesterol are co-transported to the parasite’ PV, but sterol biosynthetic pathway, or LDL defective cell lines, do not hamper parasite growth, suggesting compensatory ways to provide cholesterol to the parasite (144). Interestingly, the liver form’s PV is enriched in cholesterol and tightly associated only with the host ER. It is permeable to small solutes through channels stably maintained by host-derived cholesterol accumulated at the PV membrane, suggesting that sterols might contribute to parasite nutrient acquisition (144, 145). Then, intracellular parasites have many strategies to scavenge cholesterol, and host LDs are the essential source (Figure 1).

The two parasite ACAT enzymes, which esterified cholesterol (146, 147), are known and orchestrate the formation of LDs into the parasites (31, 34, 128, 138, 148). The parasite also hijacks host Golgi-derived vesicles by Rab14, Rab30, and Rab43 transport, small GTPases family members, within the PV and acquires their sphingolipid content (125).

In Figure 1, we summarize the essential and general ways discussed above by which parasites capture cholesterol from membrane receptors and host LDs. The organelle interactions highlight their importance on the traffic of lipids and metabolites. Moreover, the LD formation on both host and parasite cytoplasm, as well as the LD’s phagocytosis, discussed below are also illustrated as general characteristics of intracellular parasites.

Intraerythrocytic *Plasmodium falciparum* and *T. gondii* tachyzoites can synthesize neutral lipids, such as triacylglycerol, and store them in LDs present in the parasite cytoplasm (34, 148). The three forms of *T. cruzi*’s life also have intracellular LD formation. Epimastigotes cultured in media with higher concentrations of serum showed an increase in both cytoplasmic LD and LD-associated to reservosomes. In LD fraction present in purified reservosomes, the major neutral lipid was cholesterol and cholesteryl ester. No sterol synthesized by parasite pathways, such as ergosterol or ergosterol esther was found, which reinforces the idea that the lipid content of the LD-associated with reservosomes comes from the uptake of exogenous lipids (149). Once endocytosed, these lipids may be used in biosynthetic pathways of the *T. cruzi*. Carriers ABCA1-like, closely related to the mammal’s cholesterol and phospholipid carriers (150), were detected associated with the plasma membrane, intracellular vesicles and flagellar pocket in *T. cruzi* (151) and may participate in the cholesterol transport captured into the cytoplasm. Furthermore, the presence of Rab 18 in reservosomes could contribute from the traffic of lipids to other organelles (152).

Interestingly, the LDs formation in *T. gondii* cytoplasm was observed only when the host cells are in a higher concentration of lipids (34). Then, the presence of LDs on *P. falciparum* and *T. gondii* was associated with parasite energy store and homeostasis; since they are in a higher concentration of lipids, they could be toxic to these parasites (34, 148).

#### LDs in a Mechanism of Heme Detoxification

Considering that the cholesterol source for an extracellular parasite residing within the mesenteric veins and capillaries of their hosts, would not be a problem, *Schistosoma mansoni* indeed induce a reduction in serum total cholesterol of their host (153). Curiously, this capability of hijack cholesterol from its host is correlated with decrease LD formation in the host. The *S. mansoni* experimental model also shows the decreased levels of serum cholesterol, and of LDs in the liver cells of ApoE deficient mice fed a high-fat diet. Importantly, it also induced a systemic reduction of lipid since it was observed in adipose tissue around liver, heart, and blood vessels (153). The mechanisms responsible for this decrease remain unknown, but it may be dependent on the presence of parasite eggs and are related to the immune granulomas formed around them (153, 154). It was reported that eggs of *Schistosoma japonicum* require cholesteryl ester for their maturation. The uptake of these lipids came from HDL particles and CD36-related protein-mediated internalization (155).

Noteworthy, *S. mansoni* may also secrete parasite LDs. Numerous LD in the gut of parasites had hemozoin associated, and the authors suggested the role of LD in a mechanism of heme detoxification during parasite blood feeding (156, 157). In agreement with this view, other authors observed a prominent production of LD in *Plasmodium berghei*-infected hepatocytes and inside the parasitophorous vacuole and the food vacuole of *P. falciparum-*infected erythrocytes (27, 158, 159). These data suggest that the LDs might play roles in the detoxification of heme in the *Plasmodium* parasites (148). As summarized in Figure 1, the data discussed in the Sections “Protozoa Are Unable to Synthesize Cholesterol De Novo and May Retrieve It From Host LDs” and “LDs in a Mechanism of Heme Detoxification”indicate that LD may exert a parasite energy store and a functional role in parasite homeostasis including detoxification functions.

#### LDs in Parasite May Modulate Host Response

**Figure 2:**
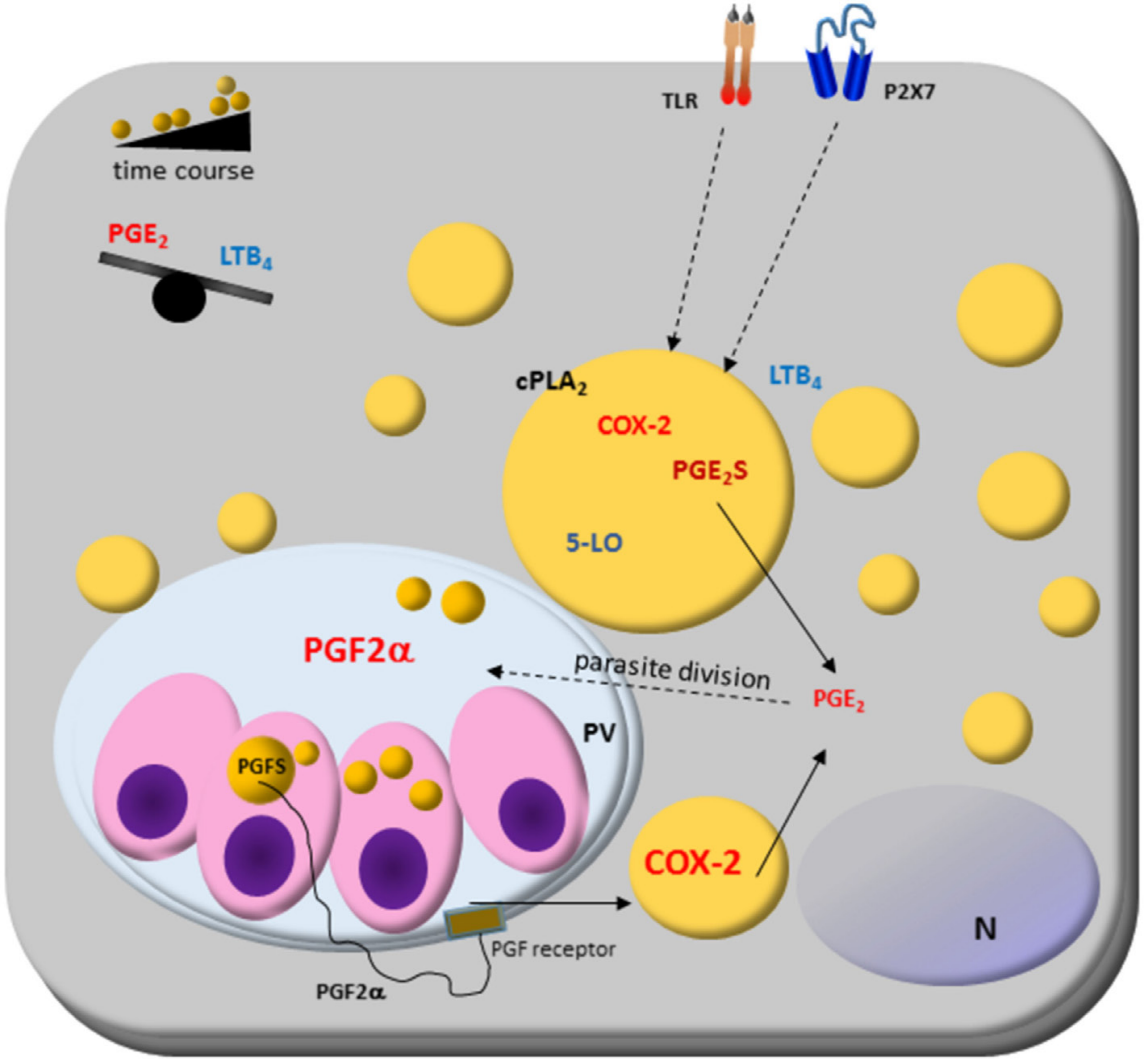
Host cell lipid droplet (LD) biogenesis in response to interaction with protozoan parasites. Increased numbers of LDs occur in the cytoplasm of many cell types after infection with different parasite-containing vesicles as well as on cytoplasm of these parasites, such as *Trypanosoma* spp., *Leishmania* spp., *Plasmodium* spp., and *Toxoplasma gondii*. The signaling from toll-like receptors (TLRs) by pathogens and pathogen-derived molecules triggers signaling pathways, such as PPARs and MAPKs, which are involved in the formation of LD. LDs produce lipid mediators; they compartmentalize both the substrate and the enzymatic machinery necessary for eicosanoid syntheses, such as cPLA2, cyclooxygenases (COX)-2, and prostaglandin synthases. Prostaglandin E_2_ (PGE_2_) production benefits parasite survival, as shown in *Trypanosoma, Leishmania, Plasmodium*, and *Toxoplasma* infections. On the other hand, leukotriene B_4_ (LTB_4_) production by host cells *via* P2 × 7 receptor is related to parasite killing as seen in *Leishmania* infection, where LDs biogenesis and an anti-inflammatory balance in the PGE_2_/LTB_4_ axis could facilitate the *Leishmania* transmissibility and infection *in vivo*. Cytoplasmic LD in *Leishmania* express prostaglandin F2α synthase (PGFS) responsible for PGF_2α_ production. The PGF_2α_ receptor (FP) is present on parasite vacuole (PV) surface, suggesting these LDs could act as a virulence factor. Then, LD could be involved in inflammation and immune evasion.

Intracellular parasites, such as *T. cruzi, Leishmania infantum chagasi, P. falciparum*, and *T. gondii*, also have cytoplasmic LDs. Notably, experiments with *T. cruzi* and *L. i. chagasi* are shedding light into LD functions in parasites. They went beyond sites for energy storage and suggested their involvement in the modulation of the host immune response, and participation in mechanisms of virulence (46, 160). These recent findings are illustrated in Figure 2 and discussed below.

Novel findings show that *L. i. chagasi* cytoplasmatic LDs are intracellular sites of prostaglandin synthesis, mostly PGF_2a_ production (45). *L. i. chagasi* has increased LD numbers and amplified expression of prostaglandin F2α synthase according to metacyclogenesis stage: metacyclic and amastigotes LD formation raised, and is the PGF_2a_ source. Accordingly, it was demonstrated that the FP receptor is localized in the early phagocytic vacuoles and on surface parasitophorous vacuoles during macrophages infection with metacyclic forms of *L. i. chagasi*. Furthermore, the inhibition of the FP receptor in the macrophages diminished the *L. i. chagasi* parasite load 72 h after infection showing that the parasites may mobilize the eicosanoid machinery in the most infective stage of the parasite and suggest that the *Leishmania’s* LD could act as a virulence factor (45).

*Trypanosoma cruzi* LDs have also been associated with eicosanoid metabolism. The LDs of amastigotes growing *in vivo* have increased the size and electron-density than those from amastigotes cultured *in vitro* (46). Trypomastigotes forms also increased LD after both host interaction and exogenous AA stimulation. The AA content in LDs purified from AA-stimulated parasites increased and these parasites released high amounts of PGE_2_ and showed PGE_2_ synthase expression. Importantly, a PGE_2_ synthesis occurred within LDs from AA-stimulated trypomastigotes, indicating that LDs are essential sources of PGE_2_, a potent lipid mediator that inhibits many aspects of immune responses, and can be implicated in the pathogen survival (46). These data demonstrate novel functions for parasite-derived LDs in eicosanoid metabolism and evasion of the host immune response (45, 46).

### Parasites Induce LDs on Host Cells: Friend or Foe?

**Figure 3:**
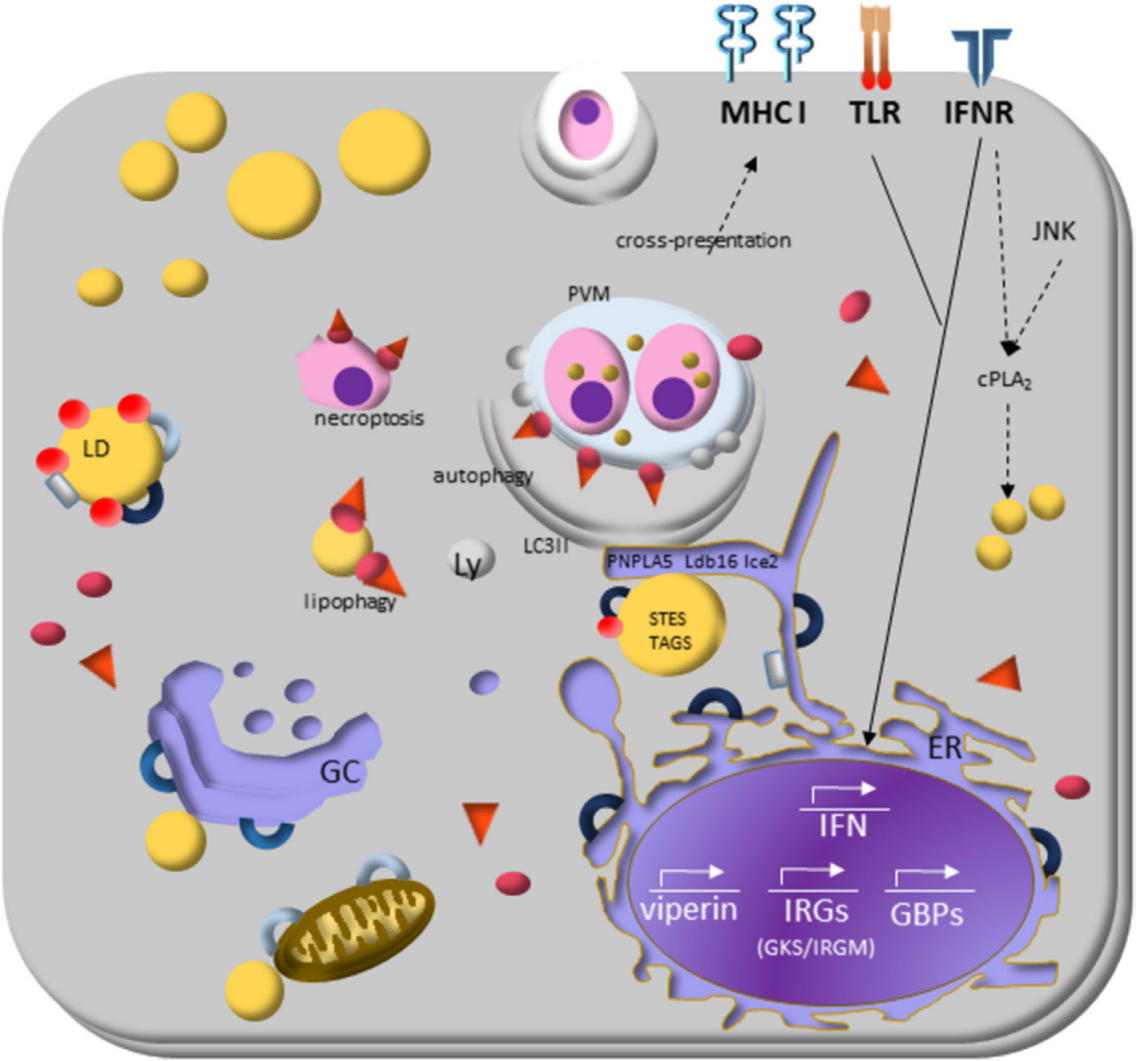
Lipid droplets (LDs) and the molecular mechanisms regulating immune response during protozoan infection. Interferon (IFN)-γ receptor signaling induces transduction of GTPases family molecules—immunity-related GTPases (GKS e IRGM proteins) and GPBs subfamilies—that play essential roles in membrane vesicular trafficking, autophagy, and antimicrobial and anti-inflammatory responses. IRGM are abundant proteins on LDs and “protects self-vesicles” from GKS and guanylate binding proteins (GBPs) destruction by autophagy machinery. The inactive GKS and GBP proteins built a pool of molecules available on cytoplasm and could have a stable association with the IRGM-deficient membrane, as parasitophorous vacuole membrane and eventually as LDs, to target them to autophagy, or lipophagy, respectively. LDs provide fatty acids, and lipids employed the phagophore membrane, implying their enzymes [steryl esters (STEs) and TAGs] on autophagosome biogenesis. IFN-γ signaling induces upregulation of LDs, and it appears to involve perilipin 2 (PLIN2)/ADRP that physically associates with IRGM. LD is also related to cross-presentation pathway since depletion of LDs by pharmacological inhibition of DGAT and by PLIN2/ADRP deficiency led to decreased LD biogenesis and cross-presentation since MHC-I surface expression, and the rate of antigen-presenting cell conjugated with T CD8+ lymphocyte are reduced.

Increasing data about the modulation of host lipid metabolism through targeting LD formation and accumulation by intracellular pathogens emerge as a typical phenotype to viral, bacterial, and parasitic infections and highlights the involving of LD in different aspects of inflammation [reviewed by Bozza et al. (5) and Saka and Valdivia (17)]. However, the understanding of LD formation and their dynamics in the interplay between hosts and parasites is still limited. Here, we will discuss the mechanisms known that parasites induce LD formation on host cells. Figures 2 and 3 summarize the data knew until now.

#### LDs in Eicosanoids Production and the Regulation of Host-Parasite Interactions

As a major source of eicosanoids production, LDs have been implicated in the regulation of parasite-induced inflammation [reviewed in Bozza et al. (5, 90)]. Eicosanoids are bioactive molecules produced by the oxidation of AA by specific enzymes, such as cyclooxygenases (COX), lipoxygenases, and terminal eicosanoid synthases. Prostaglandins have a wide variety of activities, including down-regulation of macrophage functions, and regulatory roles in immune responses. Notably, COX-2 and PGE-synthase compartmentalize within LDs (10, 18, 21), and LDs were the main site for heightened PGE_2_ production during *T. cruzi* infection (30).

The crucial role of PGE_2_ in the modulation of the host immune response can be illustrated by its higher plasma levels in patients with localized or diffuse cutaneous leishmaniasis than patient control (161). Also, the PGE_2_ secretion through PLA_2_ induced in the progression of cutaneous disease in *L. amazonensis* infection (162). More than that, PGE_2_ production benefits parasite survival, as showed in *Leishmania* spp. (35, 163–165), *T. cruzi* (20, 30, 166), and *T. gondii* infections (32, 33, 37).

This association between LD formation and PGE_2_ production was observed during *in vivo* infection with *T. cruzi* (20). Infected macrophages exhibit positive immunostaining for COX-2 and PGES, and both co-localize with PLIN2/ADRP staining, a LD structural protein, which confirms the presence of these enzymes inside LD in *T. cruzi*-infected macrophages. The association of PGE_2_ production and parasite division highlighted the LD participation on evasion mechanisms of *T. cruzi* (30, 167).

In *Toxoplasma*-infected macrophages and muscle cells, the host LD formation is associated with reduced host microbicides properties, with an increased PGE_2_ production, a potent inhibitor of Th1 response, and with a decreased nitric oxide production (32, 33). In muscle cells, the number of LD increased in a time course manner earlier as 6 h, until 48 h post-infection. The LD formation correlated with higher COX-2 and PGE_2_ expression, which could be contributing to IL-12 and interferon (IFN)-γ secretion from muscle cells (33). The production of the PGE_2_ with IL-12 and IFN-γ seems contradictory. However, IFN-γ is essential to control *T. gondii* infection—and the infections with others parasites [reviewed in Ref. (168–170)].

The secretory serine protease of *Leishmania donovani* is involved in down-regulation of macrophage microbicidal activity by inducing COX-2-mediated PGE_2_ release (171). The cytokines, such as IL-12 and IFN-γ, could favor the cyst formation in muscle cells and the establishment and maintenance of the chronic disease and transmission source *via* consumption of raw or undercooked meat containing *T. gondii* (33). The mechanisms of these processes await further investigation. In dendritic cells, *L. amazonensis* infection likewise induced LD formation and the modulation of cholesterol uptake pathways (172). Differently, *L. i. chagasi*-infected macrophages did not show LD formation, only the parasites showed LD formation (45).

The pathogenesis of severe malarial anemia is also due to the release of soluble mediators of inflammation as part of host immune response driven by phagocytosis of malarial pigment (hemozoin) present in parasitized RBC or free release on lysis of these cells (173). Although the suppression of COX-2-derived PGE_2_ is also associated with enhanced severity of malaria, how it alters the erythropoietic cascade remains to be determined (174, 175). Recently, increased LDs were demonstrated during malaria infection in different mice strains, however, there are not an association of number of LDs and disease severity (28).

Importantly, the stimulation of PLA_2_-COX-2-PGE_2_ pathway that can suppress macrophage’s activation is observed only in macrophages infected with live *L. major* parasites (37), highlighting the active role of the parasite with its cell host. Nevertheless, the protein level of COX-2 is unchanged in *L. donovani* (176) and *L. major* infection, suggesting that LD formation may not correlate with PGE_2_ production in *L. donovani* and *L. major* infections (48). However, recent data showed the essential role of PGE_2_ in suppressing *L. major*-infected macrophages, and this suppression was mediated by B1 cell IL-10 production (177).

A key observation is the host LDs in close apposition—and even inside—parasite-containing phagosomes, such as in *T. cruzi* (20, 49), *L. major* (48), *L. amazonensis* (172), and *T. gondii* infections (33, 34). Nolan et al. (34) showed that the host LDs are necessary for *Toxoplasma* infectivity in human foreskin fibroblasts (HFFs) and mouse embryonic fibroblasts (MEFs). The host LDs progressively increased with a peak of 8 h post-invasion (p.i.) that coincided with PLIN2/*ADRP* and *GDAT2* upregulation transcription, suggesting stimulation of TAG synthesis in LD, and the host LDs cluster around PV. After that, their number decline and abruptly dropped until parasite egress at 24 h in HFF. In MEF, the host LD and GDAT2 decrease but the LDs remain around the PV. The authors suggested that the LD biogenesis and breakdown for lipolysis, the distribution of LD, and the DGAT2 expression levels reflect the highly dynamic status of host neutral lipid metabolism during infection. *T. gondii* may drive it, but it is also be part of a host cell defense mechanism in response to parasite-mediated lipid imbalances (34).

Lipid droplet formation in *L. major*-infected macrophages was very quick, time-dependent, and independent of parasite viability. It suggests that the trigger for LD formation by *L. major* also has a cellular origin, as *T. cruzi* and *T. gondii* infections do (30, 33, 37). Microarray data and transcriptomic analysis from *L. major*-infected macrophages support this formation. They revealed an enhanced cholesterol-uptake coupled with a decreased cholesterol efflux (48) and cholesterol uptake combined with activation of *de novo* TAG synthesis (37). Furthermore, LD formation was independent of cytoskeleton movement and *L. major* phagocytosis (37). As suggested for mycobacterial infections (41, 178), the paracrine induction of LD formation is also observed in the parasites *T. cruzi* (30), *Toxoplasma* (33) and*L. major* (37) infection. Then, soluble factors from infected cells or parasites may act in a paracrine manner to induce LD formation in uninfected bystander cells suggesting that receptors are triggering and downstream signaling pathways are sufficient to cause LD formation (30, 37, 178). It seems to have more and fundamental participants to achieve a homeostasis pattern. In *L. amazonensis* infection, Afonso e collaborators suggested that PGE_2_ and TGF-β1 pathways are involved with the enhanced parasite burden in *L. amazonensis* (179).

The roles of LDs in leukotriene synthesis during infection by intracellular parasites have not yet been established. This would be an important point to address since there is solid evidence that leukotriene B_4_ (LTB_4_) production by host cells is related to parasite killing in *Leishmania* infection by nitric oxide and reactive oxygen species production (180–183). Although there is evidence that 5-LO enzyme co-localize with LDs in hepatic stellate cells from schistosomal granulomas, where it is involved in the release of Cys-LTs in a TGF-β-regulated manner, potentially influencing pathogenesis and liver fibrosis in schistosomiasis (184).

The *Leishmania* vector (sand fly, *Lutzomia longipalpis*) saliva components have a central role in parasite infection. They can modulate eicosanoids metabolism and LDs formation in the host cells (164, 185, 186). The *Lutzomia longipalpis* saliva activates macrophages to produce PGE_2_ but not LTB_4_ (186) and the sand flies salivary gland sonicate (SGS) increases PGE_2_ production by neutrophils during *L. infantum* infection (164). The SGS during *L. infantum* inoculation increased the parasite viability inside peritoneal cells, and COX-2 inhibition blocked this action. The LD biogenesis on host or parasite and an anti-inflammatory balance in the PGE_2_/LTB_4_ axis could facilitate the *Leishmania* transmissibility and infection *in vivo* (160, 186).

The lipid derived pro-resolving mediators called lipoxins, resolvins, and protectins are potent anti-inflammatory mediators involved in the resolution of inflammation. As reviewed by Russell and Schwarze, lipoxins are protective to host control of disease caused by *T. gondii, T. cruzi*, and *P. berghei* but not by *Mycobacterium tuberculosis* bacteria. It could be related to the balance between pathogen-control and excessive immune response (187). However, the role of LDs in the synthesis of lipid mediators involved in the resolution of inflammation has not yet been characterized and it would be of interest to investigate whether LD is also involved in lipoxin- or resolvin-mediated mechanisms of pathogen evasion or destruction.

#### Lipid Droplets in Vesicular Interactions and the Resistance of Host to Parasite Infections

Interferons are effector key molecules involved in protective responses to viruses and intracellular pathogens [reviewed in Schroder et al. (168), Schneider et al. (169), and Pilla-Moffett et al. (170)]. They have their antimicrobial effects by a considerable remodeling transcriptional expression profile of target cells bearing the IFN receptors (188, 189). One of these IFN-stimulated genes encoding viperin (for virus inhibitory protein, ER-associated, and IFN-inducible), a canonical protein evolutionary conserved and found to be highly upregulated in response to LPS, dsDNA, and RNA analogs, and in infection with various viruses. Interestingly, viperin localizes to the cytoplasmic leaflet of the ER and LD, and LDs are sites of viral assembly (190, 191), and has antiviral activity against various human viruses (192).

Saka and Valdivia (17) already draw the attention for the exciting data showing that two signaling pathway (JNK and IFN) are involved in the cPLA2 activation required for LD biogenesis (193), as well as in immunity to *Chlamydia trachomatis* (194) and *C. pneumoniae* (118). In Figure 3, we highlighted the receptors described to induce LD formation and the key molecules and pathways involved in the modulation of parasite response. Others possible partners we also discuss in the following text.

Interferon-induced resistance genes include members of two GTPase families named immunity-related GTPases (IRGs) and guanylate binding proteins (GBPs). The IRGs proteins are subdivided into GKS and GMS IRGs class. The current model of IRGs interaction describes that, in IFN-γ-activated cells, the effector GKS proteins are cytosolic and stay in the inactive GDP-bound state by GMS regulatory proteins (195, 196). The specific membrane targeting of the GMS proteins prevent accumulation of activated GKS proteins and enable GKS proteins to distinguish organelles membranes from that of pathogen vacuoles. The GMS proteins are tightly associated with endomembrane of cellular organelles and restricted to specific organelles: Irgm1 localizes mainly to the Golgi complex membrane, endolysosomal compartment, mitochondria, peroxisomes, and LDs in uninfected cells; Irgm2 localizes to the Golgi and Irgm3 to the ER and LD (197).

Several members of both GTPases families can recognize specific host lipid molecules, to translocate and to adhere precisely to parasitophorous vacuole membranes (PVMs), labeling it for disruption or delivery to lysosomes to inhibit intracellular pathogen growth [reviewed in MacMicking (189)]. The specificity of these intracellular targeting events is well documented (168, 197–199) and the underlying mechanism is beginning to be deciphered (200–202). When GMS proteins are absent in the cell, GKS proteins activate spontaneously and form aggregate-like structures, preferentially on PVMs. The consequence is the disruption of the vacuole and the release of the pathogen into the cytosol or both vacuole and parasite disruption, resulting in either necroptosis or autophagy (203, 204).

Bougnères et al. (205) showed the Irgm3 role in LD formation, as Irgm3-deficient DCs do not accumulate LDs (205). DCs isolated from Irgm3-deficient mice showed the impaired capacity to stimulate specific CD8+ T cells owing to antigen phagocytosis and did not increase LD biogenesis upon IFN-γ stimulation when compared with wild-type mice. The increased antigen cross-presentation competence correlated with LD biogenesis and the chemically inhibited LD biogenesis blocked it. The data show that DCs from PLIN2 (ADRP) deficient mice had similar defects in antigen cross-presentation that Irgm3-deficient mice had and that Irgm3 interacts with PLIN2 suggest that LDs play a significant role in regulating cross-presentation (205). The absence of Irgm3 protein can explain the lack of LD biogenesis, where unprotected by IRGM protein, LD suffer an attack of GKS-GBP proteins bounding on their membrane delivering them to autophagy pathway (201).

The essential functions of GBPs family proteins seem to be their ability to target membranes and to oligomerize in them. Several GBPs harbor C-terminal CaaX motifs for isoprenylation that enable them to associate with intracellular endomembranes (206). They bind and hydrolyze guanosine phosphated, allowing GBPs to form homo- and hetero-oligomers. Once docked on PV membrane, GKS and GBPs proteins can recruit antimicrobial protein complexes, including autophagic cascade and inflammasome pathway to destroy the parasite vacuolar compartment [reviewed by Schroder et al. (168)]. Interestingly, GBP proteins stimulate caspase-11-dependent pyroptosis in macrophages infected with Gram-negative vacuole resident bacteria leading the activation of the non-canonical inflammasome (207, 208).

As inactive proteins, GKS and GBP have lipid binding substrates on LD and *T. gondii* PVMs. Haldar et al. (201) proposed that the high expression of GMS proteins on LDs make them regulatory proteins by increasing the availability of GKS and GBP inactive proteins allowing them to bind on PVMs and deliver them to autophagosomes for parasite degradation. It was recently shown that murine GBP2 positively regulates the recruitment of Irga6 (a GKS protein) to *T. gondii* PVMs (209) and directly targets the membrane of *T. gondii* after disruption of PVM (202). Then, the vacuolar contents released into the cytosol may be removed through autophagy (198, 203). The vacuolar contents released may be ubiquitinated, resulting in the recruitment of p62. ATG3 and p62 also promote the cross-presentation of vacuolar antigens, derived from lysed vacuoles, on MHC class I (210).

Haldar and colleagues showed that Irgm1 and Irgm3 co-localize on LD of IFN-γ-treated MEFs and that Gbp1 effector protein p62 co-localizes with IRGM-deficient LDs MEFs. Ichimura and Komatsu showed that p62Gbp1 binds directly to the LC3 to deliver its cargo to autophagosomes (211). As LDs from IRGM-deficient mice were inside autophagosomes upon IFN-γ activation, it could suggest that IRGM was not a role in LD formation. Instead, Irgm1 and Irgm3 proteins could protect LDs from degradation in MEFs and p62Gbp1 could start LDs lipophagy (201).

Lipophagy occurs when lysosomes degrade LD after its sequestration by autophagosomes. The recognition of LD by autophagic machinery is unknown [reviewed by Martinez-Lopez and Singh (212)]. Lipophagy could be related to lipid homeostasis, where control size and the number of LDs are making a significant balance of energy source and survival mechanisms, such as cell stress. Accumulating evidence suggest the participation of LD in the regulation of the autophagic process and their destruction by autophagy *via* lipophagy (212–214). LDs also act in concert with the proteasomal and autophagic pathways of protein degradation, proposing their role on isolation stress products inside LD, such as apo B in hepatocytes and a-synuclein in Parkinson’s disease (215, 216). On atherosclerosis, however, it seems that IRGM has a pathogenic role. IRGM proteins participate in the positive feedback where ox-LDL up-regulates IRGM in macrophage, which, in turn, modulates the CD36 to promote the ox-LDL uptake (217). Studies on other systems are necessary to investigate the IRGM pathogenic role.

Curiously, IRGM proteins physically interact with the central autophagy regulators and make molecular complexes with the pathogen sensor NOD2. In response to PAMPs, the RIG-1, and TLR3 signaling boost the NOD2-IRGM association. NOD2-IRGM complex promotes the assembly of autophagy regulators highlighting the critical role of IRGM proteins as organizer of the core autophagy machinery and as a vital component of antimicrobial and anti-inflammatory responses (200, 218, 219).

Recently, Shpilka and collaborators suggested that LDs provide fatty acids and lipids for the formation and elongation of the phagophore membrane (220). This work also showed the involvement of the enzymes responsible for the TAG and steryl esters synthesis with autophagosome biogenesis in yeasts strains. Moreover, they showed the essential participation of ER-LD contact-site proteins, Ldb16 and Ice2, in autophagosome formation. They suggested that these two organelles act together, and the lipid transfer from LD to the ER is necessary for autophagosome formation (220). Previously, the contribution of TAG mobilization from the LDs, by the lipase PNPLA5, to autophagosome biogenesis was shown (213).

As the autophagic machinery is also implicated in the formation and growth of LDs (221), feedback between these two processes could drive LD growth, autophagosome biogenesis, and cellular homeostasis.

Our current understanding of IFN-inducible GTPases role on host resistance to the intracellular protozoa and on the LD role to self-organelle recognition appoint to LD as important cell function modulator, implying the participation of LDs on parasite immune response.

## Final Remarks and Future Perspectives

A number of key findings have evidenced a protagonist role for LDs in host parasite biology. LDs were shown to have major roles in metabolism, signaling and detoxification, and actively participates in innate immunity response to infection. Parasite-host interactions modulate the lipid metabolism of both organisms, and these studies provide valuable knowledge on cell biology, immune response, and drugs development. In leukocytes and other cells involved in infectious conditions, LD biogenesis is highly induced through regulated mechanisms that involve innate immune receptors and transcriptional dependent and independent pathways. Notably, it is now well established that LDs are main sites for the generation of lipid mediators involved in the host immune responses. The evolving understanding of the interplay of LDs and critical pathways on immune response, as IFN-induced resistance genes, autophagy, antigen cross-presentation and areas that still need to have roles of LDs investigated like resolution of inflammation, could illuminate our knowledge of the signaling ways that control parasite infections or, better to say, establish homeostasis with host and parasite living together. Furthermore, it is not a surprise that the intracellular parasites have developed strategies to evade or use LDs for surviving. Parasites have adapted to exploit LDs as a source of lipids for membrane building, a crucial step for parasite growth, and as a negative regulator of immune response.

Although great advances in the understanding of the mechanisms of LD biogenesis and its roles in lipid metabolism and inflammatory mediator production have been achieved, critical questions remain about the formation and the functions that LDs play in infectious diseases. In conclusion, recent studies have identified LDs as multifunctional organelles with key functions in lipid storage and cell signaling in inflammation, and as such, they are emerging as attractive target candidates for therapeutic intervention. Future studies will be necessary to characterize the role of LDs as targets for therapeutic intervention in infectious diseases that progress with increased LD accumulation. These will need to include the development of selective LD inhibitors. Moreover, the safety characterization of LD inhibition is required, as lipid accumulation within LDs may act as a protective mechanism in lipid homeostasis against cellular lipotoxicity.

## Author Contributions

AV and PB contributed conception and design of the review. AV, KO, and LT organized the database. AV wrote the first draft of the manuscript. AV, PB, CM-M, KO, and LT contributed to writing a subset discussion and to manuscript revision, read, and approved the submitted version.

## Conflict of Interest Statement

The authors have no other affiliations or financial involvement with any organization or entity with a financial interest in or financial conflict with the subject discussed in the manuscript.
